# Novel cochlear implant assessment tool: Comparative analysis of children and adults

**DOI:** 10.3389/fneur.2023.1090184

**Published:** 2023-04-27

**Authors:** Fernanda Ferreira Caldas, Byanka Cagnacci Buzo, Bruno Sanches Masiero, Alice Andrade Takeuti, Carolina Costa Cardoso, Thais Gomes Abrahão Elias, Fayez Bahmad

**Affiliations:** ^1^Department of the Faculty of Health Sciences, University of Brasilia, Brasília, Brazil; ^2^Brasiliense Institute of Otorhinolaryngology, Brasília, Brazil; ^3^Cochlear Latin America, São Paulo, Brazil; ^4^Department of Communications, School of Electrical and Computer Engineering, University of Campinas, Campinas, Brazil

**Keywords:** cochlear implant, speech perception, audiometry, adults, children

## Abstract

**Objectives:**

To analyse the results of children and adults with cochlear implants (CIs) in pure tone audiometry (PTA) and speech perception tests. Tests were performed in two ways: using loudspeakers in the sound booth (SB) and with direct audio input (DAI) employing the *Cochlear Latin America BOX* (CLABOX).

**Methods:**

Fifty individuals (33 adults and 17 children) participated in the study, including children aged between 8 and 13 years; of these, 15 users had bilateral CIs, 35 had unilateral CIs, and all had severe to profound bilateral sensorineural hearing loss. All participants were evaluated in the SB with loudspeakers and the CLABOX with DAI. The following evaluations were conducted: PTA, speech recognition tests with the *hearing in noise test* (HINT).

**Results:**

The results for PTA and HINT conducted in SB and with CLABOX presented no significant difference between children and adults.

**Conclusion:**

The CLABOX tool presents a new possible method to evaluate PTA and speech recognition tests in adults and children, with results comparable to the conventional evaluation in the SB.

## 1. Introduction

Cochlear implants (CIs) are an effective and safe treatment that provides functional hearing and listening comprehension and aid in language acquisition in children. Implant placement surgery can be performed unilaterally or bilaterally, simultaneously or sequentially; to develop binaural skills, it is necessary to perform bilateral implant placement (1).

To maximize the rehabilitative benefits, including cochlear implants as part of the treatment plan, it’s crucial to consider performing this surgery at a younger age. Research suggests that children who receive cochlear implants before the age of 3.5 years show a quicker development of the desired cortical morphology and latency in the cortical P1 wave response (2). Niparko (3) studied 188 implanted children. The group of children who received CIs at less than 18 months of age had significantly better comprehension and language results than children who underwent implantation between 18 and 36 months and those older than 36 months. Most children who underwent implantation before 18 months had results parallel to their hearing peers; those who underwent implantation after 18 months of age had smaller increases in performance and greater variability in understanding and expression.

To evaluate and confirm the auditory abilities of cochlear implant users, traditional methods involve conducting pure tone audiometry (PTA) and speech recognition tests within a soundproof booth (SB) to assess skills such as speech detection and recognition. To perform these assessments accurately, the sound booth must have proper acoustic treatment to avoid wall reflections, approximating a free field condition, and minimal background noise to minimize external factors that could impact the test results (4, 5). It also requires high-quality loudspeakers.

An alternative to testing speech recognition in a SB is the direct audio input (DAI) assessment, which allows the input signal to bypass an external microphone, eliminating the oscillations of ambient noise and reverberation at the test site (6, 7).

Based on the need for new tools to assist audiologists in CI assessment and programming validation, the company *Cochlear Corporation* developed a portable box with a companion software called *Cochlear Latin America BOX* (CLABOX) to transmit the sound stimuli directly to the CI *via* DAI. In this study, we aimed to analyse PTA and speech perception tests results of children and adults using CIs performed in the SB and with the CLABOX with DAI.

## 2. Materials and methods

### 2.1. Ethics

The study was analysed and approved by the Research Ethics Committee of the Faculty of Health Sciences, University of Brasilia, under protocol 4327050. All participants and parents/guardians of the children consented to participate in the research. The study was carried out at a CI centre in the city of Brasilia, DF, Brazil.

### 2.2. Participants

Fifty individuals with CIs participated in the study (33 adults and 17 children). The children’s ages ranged from 8 to 13 years, with a mean of 9.7 years (± 0.8 years). The adults’ age ranged from 18 to 78 years, with a mean age of 32.3 years (± 5.8 years). 15 participants used bilateral CIs and 35 unilateral CIs; 38 had prelingual hearing loss, and 12 had postlingual hearing loss. Of these, all children were prelingual; of the adults, 12 were postlingual, and 21 were prelingual. All had at least 6 months of experience with CI use and were users of the *Cochlear Corporation* brand.

### 2.3. CLABOX with DAI assessment

To utilize the CLABOX with the DAI connector, it was necessary to install a driver for the audio interface Audiobox iOne-Presonus on the computer. The audio interface features a USB 2.0 connection, 24-bit resolution, and a frequency response of 20 Hz to 30 kHz, with 44.1, 48, 88.2, and 96 Hz sampling frequencies. The interface is connected to the cochlear implant through a stereo headphone output with an output impedance of 10 Ω.

The CLABOX calibration followed the same standards as the conventional audiometric calibration, according to ISO 8253 and the manufacturer’s information. The software was written with MATLAB and has an accompanying graphical user interface (GUI) written with MATLAB’s AppDesigner. The GUI had five tabs: one for the examiner to enter the individual’s data, one for PTA, one for the Ling test, one for the speech recognition test, and one for the examiner to adjust high-level preferences. The software was compiled using MATLAB as a standalone application.

All participants used the same CP910 speech processor with identical settings. The audio cable accessory was in the “Only” option. Thus, the participant heard only the test stimuli directly from the software, with ambient sounds excluded (7, 8).

### 2.4. Evaluation in the SB with free field

A MADSEN audiometer, model Itera II, SB, REDUSOM brand, serial number 8020, was used. All tests were performed in free field condition, with the loudspeaker positioned at 0° azimuth and at a distance of one metre from the participant. Features like the adaptive directional microphone (SCAN mode) were deactivated.

### 2.5. Evaluation in the SB and CLABOX

To assess speech recognition, the Brazilian Portuguese version of the hearing in noise test (HINT) was applied (9–12). The software randomly selected the presentation of the sentence lists, and the examiner manually analysed the number of correct words in each sentence presented. Analysing the sentence in noise, a minimum of 75% correct answers was expected. The tests were always performed on separate ears under two conditions:

1. Fixed noise, with a signal-to-noise (S/N) ratio of +10 dB, 65 dB(A) of speech and 55 dB(A) of noise;
2. Adaptive noise, noise presented at 55 dB(A) with variable levels of 4 dB in the initial stage and 2 dB in the final stage, that is, 4 dB increments for the first four sentences and 2 dB increments for the rest.

To assess PTA, frequencies of 250, 500, 1,000, 2000, 3,000, 4,000 and 6,000 Hz were investigated in separate ears, with a pulsatile stimulus of 1.5 Hz in the free field. The four-tone average was also used (500, 1,000, 2,000 and 4,000 Hz).

### 2.6. Statistical analysis

This study used a significance level of 0.05 (5%) and 95% confidence intervals. Nonparametric statistical tests were used (Mann–Whitney test, Wilcoxon test, McNemar test, Kappa and Spearman correlation analysis). To complement statistical significance and determine effect sizes, Cohen’s D (difference) was calculated, with values of 0.20 (small effect), 0.50 (medium effect), 0.80 (large effect) and 1.20 (very large effect). Only participants tested in the acoustic booth and with the CLABOX were analysed. This way, we obtained paired data, which were examined using the Wilcoxon test.

## 3. Results

The results of the comparison between children and adults in the SB, of the comparison of children and adults assessed by the CLABOX systems for speech recognition in the HINT, and data on tonal thresholds are presented in Tables 1 2. These results present differences between children and adults and the evaluation between the two systems; however, they cannot be considered statistically significant (value of *p*>0.05). In the speech perception test with fixed noise (S/N + 10 dB) and in the SB (*p* = 0.586), children had 81.8% correct answers, and adults had 77.2% correct answers; with the CLABOX (*p* = 0.784), the result was 88.3% for both children and adults. In the HINT test with adaptive noise, in the SB (*p* = 0.356), the values of the S/N ratio were 2.80 dB (children) and 3.79 dB (adults), and with the CLABOX (*p* = 0.769), the results were 1.73 dB (children) and 2.38 dB (adults). In the PTA results, the four-tone average in the SB was 23.8 dB for children and 22.60 dB for adults, *p* = 0.246, while with the CLABOX, the four-tone average for children was 31.3 dB and 28.9 dB for adults, *p* = 0.182. The effect size (Cohen’s d) was calculated to complement the statistical significance analysis. The values were small, and the maximum was 0.518, thus classified as medium. Thus, we confirm that the differences between these two groups of children and adults are small and not statistically significant.

**Table 1:** Comparison of SB and CLABOX in the groups of children and adults in the speech perception test - HINT.

			Average	Median	Standard deviation	Q1	Q3	No	CI	*p*-value	Cohen’s *d*
Fixed Noise	SB	Children	81.8%	80.3%	10.5%	77.3%	89.3%	15	5.3%	0.586	0.308
		Adult	77.2%	79.5%	17.5%	72.3%	91.8%	25	6.9%		
	CLABOX	Children	88.3%	90.0%	12.8%	85.0%	100%	15	6.5%	0.784	0.000
		Adult	88.3%	95.0%	14.1%	80.0%	100%	25	5.5%		
	Delta	Children	6.5%	4.8%	8.4%	3.6%	9.0%	15	4.2%	0.356	0.396
		Adult	11.1%	8.3%	13.5%	0.5%	21.7%	25	5.3%		
Adaptative Noise	SB	Children	2.80	2.60	3.68	1.08	4.08	15	1.86	0.394	0.279
		Adult	3.79	3.15	3.58	1.05	5.60	25	1.40		
	CLABOX	Children	1.73	1.37	3.68	−1.00	3.78	15	1.86	0.769	0.156
		Adult	2.38	2.13	4.56	−0.88	5.10	25	1.79		
	Delta	Children	−1.07	−1.44	2.62	−2.46	0.83	15	1.32	0.586	0.128
		Adult	−1.41	−2.35	2.72	−3.40	0.55	25	1.07		

**Table 2:** Comparison of SB and CLABOX in the groups of children and adults in the PTA.

			Average	Median	Standard deviation	Q1	Q3	No	CI	*p*-value	Cohen’s *d*
250 Hz	SB	Children	32.0	35.0	8.2	25.0	40.0	23	3.4	0.358	0.235
		Adult	30.1	30.0	7.7	25.0	35.0	40	2.4		
	CLABOX	Children	33.9	30.0	7.8	30.0	37.5	23	3.2	0.173	0.448
		Adult	30.9	30.0	6.3	30.0	35.0	40	2.0		
	Delta	Children	1.96	5.00	8.36	−2.50	10.00	23	3.42	0.361	0.163
		Adult	0.75	0.00	7.03	−5.00	5.00	40	2.18		
500 Hz	SB	Children	25.0	25.0	5.8	25.0	25.0	23	2.4	0.908	0.086
		Adult	24.5	25.0	6.0	20.0	30.0	40	1.9		
	CLABOX	Children	34.8	35.0	9.2	27.5	42.5	23	3.8	0.135	0.509
		Adult	30.8	30.0	7.3	25.0	35.0	40	2.3		
	Delta	Children	9.78	10.00	8.98	5.00	15.00	23	3.67	0.071	0.451
		Adult	6.25	5.00	7.32	0.00	10.00	40	2.27		
1 kHz	SB	Children	25.7	25.0	4.8	25.0	25.0	23	2.0	0.059	0.518
		Adult	23.1	25.0	5.0	20.0	25.0	40	1.6		
	CLABOX	Children	33.0	30.0	8.6	30.0	37.5	23	3.5	0.094	0.499
		Adult	29.6	30.0	5.8	25.0	35.0	40	1.8		
	Delta	Children	7.39	10.00	10.10	5.00	12.50	23	4.13	0.461	0.108
		Adult	6.50	5.00	7.18	5.00	10.00	40	2.22		
2 kHz	SB	Children	22.2	20.0	4.2	20.0	25.0	23	1.7	0.456	0.180
		Adult	21.4	20.0	4.7	20.0	25.0	40	1.4		
	CLABOX	Children	29.3	30.0	6.4	25.0	32.5	23	2.6	0.818	0.123
		Adult	28.6	30.0	5.7	25.0	30.0	40	1.8		
	Delta	Children	7.17	5.00	7.95	2.50	12.50	23	3.25	0.994	0.010
		Adult	7.25	5.00	7.92	5.00	10.00	40	2.45		
3 kHz	SB	Children	22.0	20.0	4.7	20.0	25.0	23	1.9	0.760	0.088
		Adult	22.4	20.0	4.9	20.0	25.0	40	1.5		
	CLABOX	Children	29.8	30.0	7.1	25.0	35.0	23	2.9	0.232	0.359
		Adult	27.6	25.0	5.4	25.0	30.0	40	1.7		
	Delta	Children	7.83	5.00	9.27	2.50	12.50	23	3.79	0.399	0.344
		Adult	5.25	5.00	6.50	0.00	10.00	40	2.01		
4 kHz	SB	Children	22.2	20.0	5.0	20.0	25.0	23	2.0	0.406	0.162
		Adult	21.4	20.0	5.1	20.0	25.0	40	1.6		
	CLABOX	Children	28.0	25.0	4.9	25.0	30.0	23	2.0	0.196	0.328
		Adult	26.5	25.0	4.7	25.0	30.0	40	1.5		
	Delta	Children	5.87	5.00	5.96	5.00	10.00	23	2.44	0.823	0.130
		Adult	5.13	5.00	5.72	5.00	10.00	40	1.77		
6 kHz	SB	Children	20.9	20.0	6.0	17.5	25.0	23	2.4	0.333	0.161
		Adult	20.0	20.0	5.2	15.0	20.0	40	1.6		
	CLABOX	Children	25.0	25.0	7.7	25.0	25.0	23	3.1	0.874	0.020
		Adult	24.9	25.0	5.5	20.0	30.0	40	1.7		
	Delta	Children	4.13	5.00	6.85	0.00	7.50	23	2.80	0.471	0.123
		Adult	4.88	5.00	5.72	5.00	10.00	40	1.77		
Four-Tone average	SB	Children	23.8	22.5	3.9	21.3	23.8	23	1.6	0.246	0.300
		Adult	22.6	22.5	3.9	20.0	24.1	40	1.2		
	CLABOX	Children	31.3	30.0	6.8	26.9	35.6	23	2.8	0.182	0.436
		Adult	28.9	28.8	4.9	25.9	31.6	40	1.5		
	Delta	Children	7.55	7.50	7.35	3.13	11.25	23	3.00	0.382	0.202
		Adult	6.28	6.25	5.78	3.75	9.06	40	1.79		

**Figure 1:**
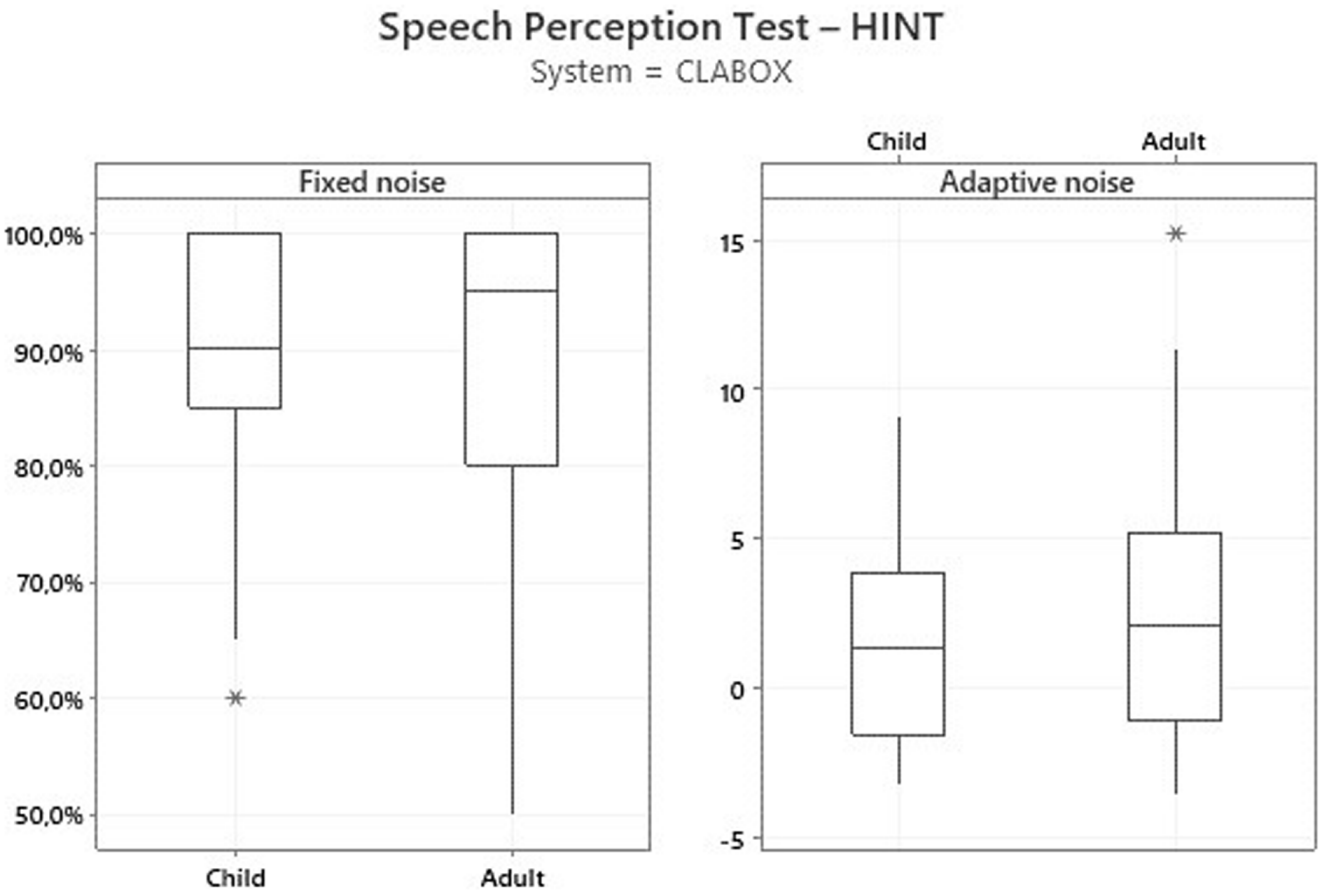
HINT with fixed noise (S/N + 10 dB) and adaptive noise at 55 dB in the SB. The box plot represents the 25th and 75th percentiles (box boundaries) and the medians (horizontal line). Outliers are indicated with asterisks.

Figures 1 2 present the results for children and adult participants in box plot format, with the HINT in fixed noise (S/N + 10 dB) and adaptive noise (noise at 55 dB) in the SB and with the CLABOX. Figures 3 4 show the PTA threshold data in the two systems when comparing children and adults. The figures with box plots represent the 25th and 75th percentiles (box boundaries) and medians (horizontal lines). The outliers are indicated with asterisks.

**Figure 2:**
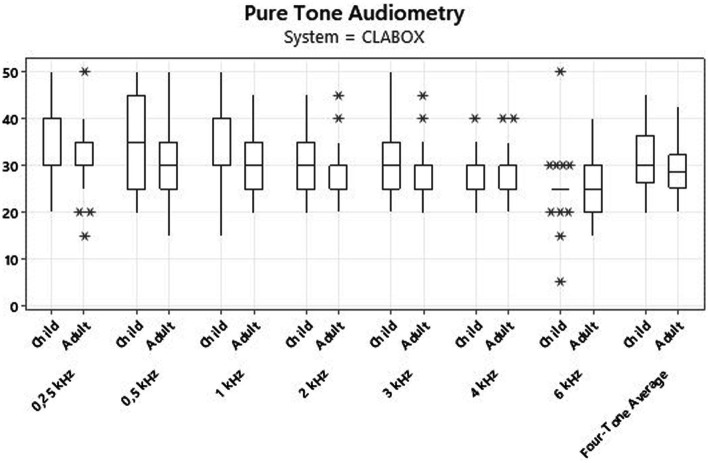
HINT with fixed noise (S/N + 10 dB) and adaptive noise at 55 dB with the CLABOX. The box plot represents the 25th and 75th percentiles (box boundaries) and the medians (horizontal line). Outliers are indicated with asterisks.

**Figure 3:**
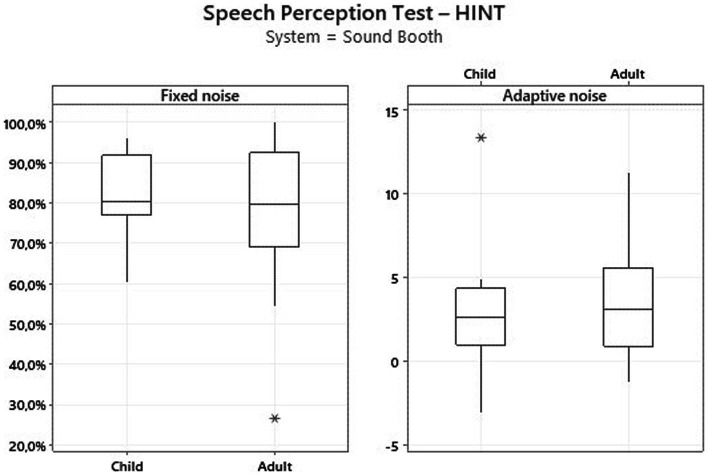
Thresholds for the PTA assessment in the SB. The results were not statistically significant at all frequencies evaluated. The box plot represents the 25th and 75th percentiles (box boundaries) and medians (horizontal lines). Outliers are indicated with asterisks.

**Figure 4:**
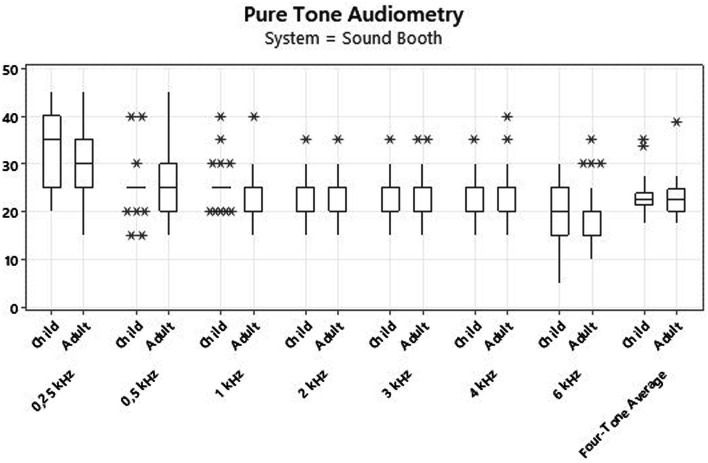
Thresholds for the PTA assessment with the CLABOX. The results were not statistically significant at all frequencies evaluated. The box plot represents the 25th and 75th percentiles (box boundaries) and medians (horizontal lines). Outliers are indicated with asterisks.

Table 3 shows an analysis of the ears of all participants who could not perform the speech perception test in fixed noise (R/S + 10 dB) using the CLABOX and SB. In the SB assessment, there were 30 participants with CI on the right ear (26.7% were children and 73.3% were adults) and 27 on the left (29.6% were children and 70.4% were adults). In the CLABOX assessment, there were 32 participants with CI on the right ear (31.3% were children and 68.8% were adults) and 28 ears on the left (32.1% were children and 67.9% were adults).

**Table 3:** Characterisation of participants by number of ears that were unable to perform the HINT.

		Fixed noise – SB		Fixed noise – CLABOX	
		Right ear	Left ear	Right ear	Left ear
Total		30	27	32	28
Children	N	8	8	10	9
	%	26.7%	29.6%	31.3%	32.1%
Adult	N	22	19	22	19
	%	73.3%	70.4%	68.8%	67.9%

## 4. Discussion

The new CLABOX tool with the DAI connector allowed us to conduct practical evaluations compared to the SB’s conventional evaluation. This would mainly be useful for centres that still do not have a way to evaluate and validate CI programming, as it is a small, light and easy-to-carry tool that can be used on a table.

Pure tone and speech perception tests were carried out on 50 participants (63 ears), including children and adults, and were performed only in the face-to-face condition. The evaluation with the DAI connection does not retain interference with background noise, room acoustics or reverberations, either in person or remotely (5, 6). The limitation of this study was that it was carried out in the first year of the coronavirus pandemic (COVID−19), which reduced the size of the sample.

Of the studies that used the connection by DAI and the SB in speech recognition tests (4, 6, 8, 13), only Goehring et al. (4) and Sevier et al. (8) collected data from adults and older children; however, the averages of the results were analysed together. This study presents speech recognition and PTA data from children and adults in separate groups.

In the evaluations between the CLABOX and SB tools, the results showed no significant differences in the PTA and speech perception tests in noise between the groups of children and adults. Sevier et al. (8) and de Graaff et al. (13) compared the results with the DAI connection in telepractice and SB using speech perception tests in silence and in noise and found similar results between the two forms of evaluation in silence; however, with the presence of noise, the modality with DAI presented better results.

The fact that children and adults have similar hearing performance can be explained by several factors, such as early implantation in children, effective use of the electronic device, and the active and effective participation of all families and/or guardians of these children in the hearing (re)habilitation process. The study by Sharma et al. (3) revealed that the central auditory system has greater plasticity in the first years of life; thus, children who undergo implantation in this period have improved cortical auditory development and the ability to respond to sounds months after implantation. Early intervention for hearing loss, centred on the family, takes place in partnership between families and professionals and is characterised by reciprocity, mutual trust, respect, honesty, shared tasks and open communication. Monitoring the evolution of listening and language skills is guided by the evolution of the child and the family (14).

In the study by Sbompato et al. (10), with normal-hearing children between 7 and 14 years of age, to assess speech perception, the results were worse in the HINT assessment when speech and noise were in the same position, that is, at 0° azimuth from the box, the S/N ratio was −3.20 dB. Novelli et al. (9) also evaluated normal-hearing children aged 8 to 10 with the HINT and found an average S/N ratio with frontal noise of −2.61 dB. In this study, the tests were performed at 0° azimuth, with S/N ratio values of 2.80 dB for children and 3.79 dB for adults in the SB and 1.73 dB in children and 2.38 dB in children. Adults had the best results with the CLABOX; however, there was no significant difference. A difference between these studies occurred in the criterion of the percentage of correct answers in the sentences; in this study, the assertive results were selected with a more difficult percentage of 75% versus 50% in the other studies (9, 10). This shows that we established a more difficult criterion for the S/N ratio and in the difference between normal-hearing children and those with CIs (15).

Regarding the results in the adult group, in the standardisation of the HINT with 13 different languages, the test with the presence of noise in the frontal position was also more difficult than that in the other conditions, and the results were similar between the languages, with an average of −3.9 dB S/N (16). In this study, we had results with a positive S/N ratio in the speech-to-frontal noise tests in the adult population. In the study by Goff et al. (15), the HINT with adaptive noise had an average of 5.87 dB and a fixed average of 71.19%; thus, these data corroborated our results. In the SB, we found 81.8% on average for children, 77.2% for adults and 88.3% for children and adults using the CLABOX. Maurer et al. (17) evaluated the speech recognition of subjects with CIs and divided them into two groups according to the responses obtained: Group 1 had a good performance, indicated by speech recognition scores between 90 and 100%, and Group 2 had poor performance with scores between 0 and 85% (17).

The studies that compared speech recognition between the tests with DAI and SB’s connection were conducted in silence and in noise (4, 6, 8, 13). In this study, we also evaluated speech recognition in noise and included the assessment of PTA; the results revealed no significant difference between the groups of children and adults. The SB had a four-tone average of 23.8 dB for children and 22.60 dB for adults, while WITH the CLABOX we observed 31.3 dB for children and 28.9 dB for adults.

The HINT measures the sentence recognition threshold, which is defined with the presentation in silence or in noise (S/N) for a listener to recognize the sentences; however, when the test is performed with CI users, some listeners may be unable to repeat the entire sentence, even in a silent condition (18, 19). Thus, some participants were not able to perform the HINT. In the SB, we had 30 ears on the right side, 27 ears on the left side, 32 ears on the right side and 28 ears on the left side. This fact can be justified by the difficulty in speech discrimination and recognition and not by the form of evaluation between the connection by DAI and the SB. This analysis may help one reach the conclusion that the CLABOX is a new assessment tool for speech recognition in individuals with CIs, both adults and children. The CLABOX tool may also be a possible option for use in countries that are starting or expanding CI indications due to its practicality of evaluation and cost-effectiveness.

In conclusion, the CLABOX tool proved to be a new assessment possibility in PTA and speech recognition tests, for adults and children, compared to the conventional assessment in the SB.

## Data availability statement

The original contributions presented in the study are included in the article/supplementary material, further inquiries can be directed to the corresponding author.

## Ethics statement

The studies involving human participants were reviewed and approved by Ethics Committee of the Faculty of Health Sciences, University of Brasilia. Written informed consent to participate in this study was provided by the participants' legal guardian/next of kin.

## Author contributions

FC conceived and planned the experiments and wrote the manuscript with support from AT, CC, and TE. BB and BM were responsible for developing the CLABOX tool. FB supervised and reviewed the manuscript. All authors contributed to the article and approved the submitted version.

## Conflict of interest

BB was employed by company Cochlear Latin America.

The remaining authors declare that the research was conducted in the absence of any commercial or financial relationships that could be construed as a potential conflict of interest.

## Publisher’s note

All claims expressed in this article are solely those of the authors and do not necessarily represent those of their affiliated organizations, or those of the publisher, the editors and the reviewers. Any product that may be evaluated in this article, or claim that may be made by its manufacturer, is not guaranteed or endorsed by the publisher.
